# Study of Natural Cytotoxicity Receptors in Patients with HIV/AIDS and Cancer: A Cross-Sectional Study

**DOI:** 10.1155/2016/2085871

**Published:** 2016-06-13

**Authors:** Orlando Nascimento Terra Junior, Gabriel de Carvalho Maldonado, Guilherme Rohem Alfradique, Vinicius da Cunha Lisboa, Adriano Arnóbio, Dirce Bonfim de Lima, Hilda Rachel Diamond, Maria Helena Faria Ornellas de Souza

**Affiliations:** School of Medical Sciences, Rio de Janeiro State University, Professor Manoel de Abreu Avenue 444, 20550-170 Rio de Janeiro, RJ, Brazil

## Abstract

The NCR receptors play a fundamental role in the cytotoxicity mediated by NK cells against tumor cells. In the current study, we investigated possible HIV/AIDS-related changes in the expression of the NCR receptors comparing healthy donors, HIV/AIDS patients, and HIV/AIDS patients with cancer (HIV/AIDSWC). The NCRs were quantified in NK cells (NK^dim^ and NK^bright^) and T lymphocytes from peripheral blood samples by flow cytometry. We found a significant decrease in the frequency of NK cells expressing NKp46 in HIV/AIDS group (*p* = 0.0012). There was a decrease in the frequency of NK cells expressing NKp46 in the HIV/AIDSWC group; however, this was not statistically significant. We found a significant decrease in the frequency of NK cells expressing NKp30 in the HIV/AIDS group (*p* = 0.0144). There was a decrease in the frequency of NK cells expressing NKp30 and in the HIV/AIDSWC group, but this was not statistically significant. There were no changes in the distribution of NK cells and their subtypes in both groups.

## 1. Introduction

HIV-1 infection is characterized by a decline of CD4^+^ lymphocyte levels and systemic immune hyperactivation [1]. These factors, when associated, lead to exhaustion of immune resources and an increased risk of cancer [2], such as Kaposi's sarcoma (KS) caused by human herpes virus 8 (HHV-8), the non-Hodgkin lymphomas (NHL), some of which are caused by Epstein-Barr virus, and cervical cancer caused by oncogenic subtypes of human papillomavirus (HPV) [3]. These tumors are known as AIDS-defining cancers by the Disease Control and Prevention Center (CDC) [4]. People infected with HIV-1 also have an increased risk of a number of non-AIDS-defining cancers [2, 4], including some associated with coinfections or cigarette smoke [5, 6].

NK cells are a subset of lymphocytes that are capable of eliminating malignantly transformed or infected cells. This population of cells is primarily in peripheral blood and bone marrow, although it can be found in secondary lymphoid organs [7]. There are two distinct subtypes of NK cells based on the density of the surface expression on CD56. Over 90% of NK cells belong to the CD56^dim^ subtype, which has as its main function cytotoxicity and is rich in granzyme and perforin [7]. The second subtype, CD56^bright^, is rare in the blood (±10%) and is responsible for the production of cytokines [7].

The precise mechanisms through which NK cells recognize and eliminate malignant or virus infected cells are complex and still not fully understood [8]. Unlike other lymphocytes, NK cells do not have receptors for specific antigens. Their cytolytic activity and cytokine production are regulated through activation or inhibition of receptors on their surface [7]. These receptors compose distinct families of proteins: lectin-like domains (CD94/NKG2A, HLA-E ligand with inhibitory function and NKG2D, and MIC-A ligand with activating function), immunoglobulin-like domains (KIR), and natural cytotoxicity receptors (NCR), where NCRs represent a family of characteristic NK cell markers [8].

There are three types of NCR receptors: NKp46, Nkp44, and NKp30. These receptors play a fundamental role in the cytotoxicity mediated by NK cells against tumor cells, whereas there is a close correlation between the expression density of the NCR on NK cells and their ability to kill tumor targets [9–11]. However, the ligands of these receptors in tumor cells have not yet been identified [8].

Previous studies have observed changes in the NCRs in HIV-1 infection [11–13]. These changes play a crucial role in some neoplasms mainly in cases of immunodepression where these receptors tend to be underexpressed [12, 14].

From a cancer perspective, prior studies observed changes in expression of NCRs in patients with malignancies, which in part is explained in the tumor microenvironment that is capable of overactive certain inflammatory responses by secreting proinflammatory cytokines that allow increased expression of these receptors [15, 16]. However, studies that observe functional changes of the NK cells in patients with both conditions are scarce.

The role of cancer in changing the profile of these cells in HIV/AIDS or if the prevalence of cancer in these groups is directly related to changes in these receptors given their crucial role in the monitoring of malignant cells is still uncertain.

The aim of this study was to evaluate the expression of NCR receptors in the NK cells (and their subtypes) and to compare HIV/AIDS patients and HIV/AIDS patients with cancer (HIV/AIDSWC). This study offers new unexpected viewpoints of the role of NK receptors and their possible exploitation in a growing range of diseases, like cancer.

## 2. Methods

### 2.1. Study Design

This was an observational study that used a cross-sectional approach conducted from 2014 to 2015 at the Department of Infectious and Parasitic Diseases of the Pedro Ernesto University Hospital, Rio de Janeiro State University, RJ, Brazil.

### 2.2. Study Subjects

The patients (groups HIV/AIDS and HIV/AIDSWC) were recruited at the Department of Infectious and Parasitic Diseases, Pedro Ernesto University Hospital, Rio de Janeiro State University, RJ, Brazil. HIV-1 diagnosis was made through a standard screening enzyme-linked immunosorbent assay (ELISA; Elecsys® HIV combi PT) and confirmed by Immunoblotting (Imunoblot Dual Path Platform (DPP®) HIV-1/2).

The inclusion criteria for the patient groups included the following: confirmed diagnosis of HIV/AIDS formally registered in medical records; age of 18 years or more; and having started antiretroviral treatment (HAART); and for the HIV/AIDSWC group they included confirmed diagnosis of cancer registered formally in medical records and being treated against cancer.

The exclusion criteria included the following: opportunist infections within 6 months, diabetes, and autoimmune diseases. To ensure that such conditions were not included in the groups, an analysis of the medical records of all patients captured for study was conducted. There were no exclusion criteria from the point of view of the medicines used for the treatment of HIV/AIDS and cancer.

The samples from the groups HIV/AIDS and HIV/AIDSWC were obtained from peripheral blood at the Nucleic Acids Laboratory of the Pedro Ernesto University Hospital, RJ, Brazil.

Healthy donors were recruited at the Department of Pathology and Laboratory, School of Medical Sciences, Rio de Janeiro State University, RJ, Brazil. For the recruitment of healthy donors, an interview was conducted, and when there was no declaration of infections within 6 months, cancer, diabetes, immunologic illness, or current use of immunomodulatory medications, blood samples were collected. A second screening was performed based on laboratory examinations and clinical records, and individuals presenting anomalies and other conditions that could affect the immune system were excluded.

All samples (5 mL of peripheral blood) were collected into a tube containing ethylenediamine tetraacetic acid (EDTA).

### 2.3. Ethics

This study was approved by the Ethics Committee of the Hospital Universitário Pedro Ernesto/UERJ (CAAE 14189113.2.0000.5259). All samples were collected with written informed consent.

### 2.4. Reagents

Anti-CD3-FITC clone UCHT1, anti-CD56-PC5 clone N901, anti-CD335-PE (NKp46) clone BAB281, anti-CD336-PE (NKp44) clone Z231, and anti-CD337-PE (NKp30) clone Z25 were obtained from Beckman Coulter (Fullerton, CA). The BD Multitest contained anti-CD3-FITC (clone SK7), anti-CD8-PE (clone SK1), anti-CD45-PercP (clone 2D1), and anti-CD4-APC (SK3) were obtained from BD Biosciences (San Jose, CA).

Anti-CD335-PE, anti-CD336, and anti-CD337 were used for the analysis of NCR family member expression. The BD Multitest was used for the quantification of the T lymphocytes CD4^+^ and CD8^+^. The anti-CD3-FITC and anti-CD56-PC5 were used for identification of the lymphocytes groups.

### 2.5. Flow Cytometry

The samples were stained with specific monoclonal antibodies (5 *μ*L of each monoclonal antibody was added to 100 *μ*L, approximately 1 × 10^6^ leukocytes of peripheral blood with EDTA) and incubated at room temperature for 15 min in the dark. The red blood cells were lysed with a FACS Lyse reagent (BD Biosciences, San Jose, CA) and centrifuged at 2000 rpm for 5 min. The supernatants were discarded, and the cells were washed twice with phosphate-buffered saline (PBS) and resuspended in 200 *μ*L of PBS. At least 200,000 events were acquired using a FACScanto II flow cytometer (BD Biosciences, San Jose, CA), and data were analyzed using the FACS Diva (BD Biosciences, San Jose, CA).

### 2.6. Gate Draw

**Figure 1:**
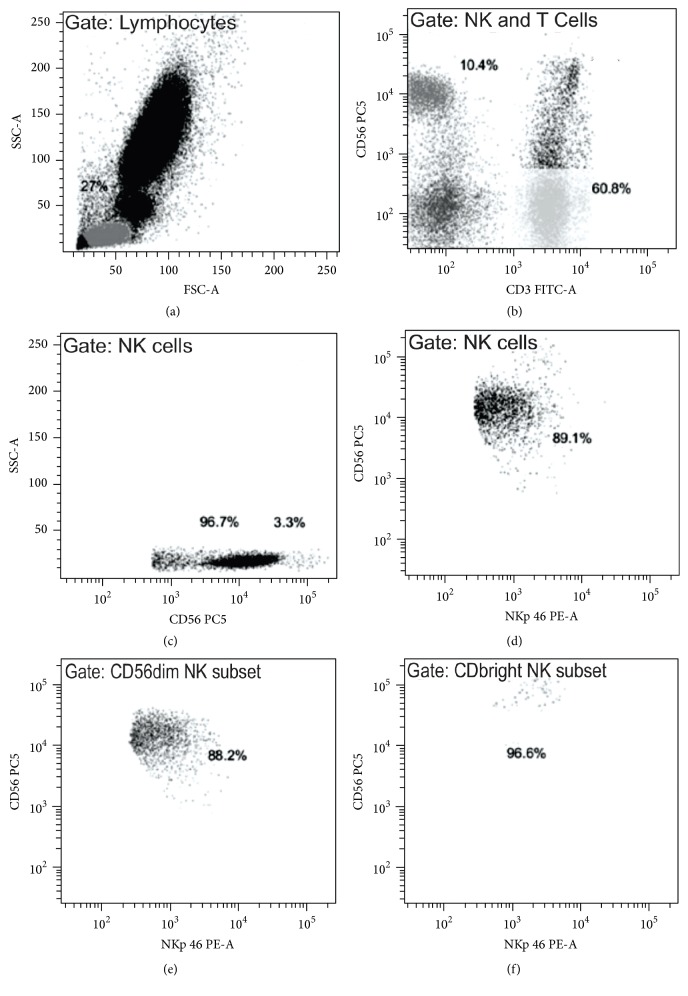
Gate strategy based on example of analysis of a healthy donor (male, age 31 years). Gate strategy: (a) lymphocyte population based on FCS and SSC. (b) Identification of cells populations based on CD3 and CD56 expression, T cells (CD3^+^CD56^−^), and NK cells (CD3^−^CD56^+^). (c) Identification of NK cell subsets based on CD56 expression. (a–c) the frequency of each cell population is represented in percentage inside each graphic. (d–f) NKp46 expression in each of these cells populations, for example, in NK cells (d), NK CD56^dim^ subset (e), and NK CD56^bright^ subset (f). (d–f) the frequency of NKp46 cells in each cell population is represented in percentage inside each graphic.

Before gate draw, the dead cells and conjugates (doublets) were excluded from the analysis. For the gate draw in the lymphocytes population, side scatter (SSC) and forward scatter (FSC) were used. The antibodies anti-CD3 and anti-CD56 (present in all tubes) were used to separate two cell populations: T cells (CD3^+^CD56^−^) and NK cells (CD3^−^CD56^+^). The NK cells were subdivided based on CD56 expression in two subsets: NK CD56^dim^ and NK CD56^bright^. Receptor expression of NCR was assessed separately for each of these populations. Gates strategies were designed based on Almeida-Oliveira et al. [7], as exemplified in Figure 1.

### 2.7. Statistical Analysis

Differences between groups were analyzed using the Kruskal-Wallis nonparametric test (with Student-Newman-Keuls posttest). Correlation analysis was performed using Spearman's test. Statistical analysis was performed using BioStat version 5.0 software.

## 3. Results

### 3.1. Baseline Characteristics of Subjects

We studied 71 subjects with ages ranging from 19 to 69 years. Our study population was divided into three groups: healthy donors (*n* = 25), HIV/AIDS patients (*n* = 25), and HIV/AIDS patients with cancer (HIV/AIDSWC) (*n* = 21). The patients' characteristics are shown in Table 1. The types of HAART are shown in Table 2.

The types of cancer that composed the HIV/AIDSWC group were Kaposi's sarcoma (8) non-Hodgkin lymphoma (8), anal carcinoma (1), fibrosarcoma (1), colon (1), mesenchymal cells (1), and Hodgkin lymphoma (1). At the time of collection, all patients were in treatment against the cancer.

### 3.2. Changes in Lymphocytes Populations

With flow cytometry, we defined the populations of interest based on CD3 and CD56 expression (Figure 1). The percentage of classical T cell population presented a slight increase in the HIV/AIDS and HIV/AIDSWC groups; however, no statistical difference was observed among the different groups nor in absolute number (Table 3). No differences were observed in the percentage of the total lymphocyte population among the different groups (Table 3).

The percentage of CD4^+^ and CD8^+^ subpopulations was affected by HIV/AIDS, as expected (Table 3). The groups HIV/AIDS and HIV/AIDSWC showed a decrease of the CD4^+^ percentage when compared with the healthy group (*p* < 0.0001). The same was found in absolute number (*p* = 0.0002). The HIV/AIDS and HIV/AIDSWC groups also showed a significant statistical difference in the CD4^+^ percentage between themselves (*p* < 0.05). This difference was also observed in absolute number (*p* < 0.05).

The HIV/AIDS and HIV/AIDSWC groups showed an increase of the CD8^+^ percentage when compared with the healthy group (*p* < 0.0001). The same was found in absolute number (*p* = 0.0007).

No differences were observed in the percentage of the NK cell population among different groups (Table 3).

The HIV/AIDS and HIV/AIDSWC groups did not show statistical differences in the WBC count when compared to the healthy group (Table 3).

### 3.3. Changes in NK Cells Subsets

No differences were observed in the percentage of the CD56^dim^ cell population among different groups (Table 3). In addition, no differences were observed in the percentage of the CD56^bright^ cell population among different groups (Table 3).

### 3.4. Changes in NCR Receptors

We investigated the expression of three receptors belonging to the NCR family: NKp44, NKp46, and NKp30. None of these receptors were expressed at considerable levels in T cells. In our study, the expression of NKp44 in the studied cells is not apparent in considerable levels.

We found a significant decrease in the percentage of the frequency of NK cells expressing NKp46 in the HIV/AIDS group (*p* = 0.0012). This difference was also observed in absolute number (*p* = 0.0018). There was a decrease in percentage and absolute number of the frequency of NK cells expressing NKp46 in the HIV/AIDSWC group; however, it was not statistically significant (Table 4). We also found a significant decrease in the percentage of the frequency of CD56^dim^ cells expressing NKp46 in the HIV/AIDS group (*p* = 0.0008). This difference was also observed in absolute number (*p* = 0.0019). The HIV/AIDS and HIV/AIDSWC groups also showed a statistical difference in the frequency of NK cells and CD56^dim^ cells expressing NKp46 in percentage between themselves (*p* < 0.05). This difference was also observed in absolute number (*p* < 0.05).

A significant decrease in percentage of the frequency of NK cells expressing NKp30 in the HIV/AIDS group (*p* = 0.0144) was observed. This difference was also observed in absolute number (*p* = 0.0011). There was a decrease in percentage and absolute number of the frequency of NK cells expressing NKp30 in the HIV/AIDSWC group; however, these results were not statistically significant (Table 4). We also found a significant decrease in percentage of the frequency of CD56^dim^ cells expressing NKp30 in the HIV/AIDS group (*p* = 0.0286). This difference was also observed in absolute number (*p* = 0.0022). The HIV/AIDS and HIV/AIDSWC groups also showed a statistically significant decrease in the frequency of NK cells and CD56^dim^ cells expressing NKp30 in absolute number between themselves (*p* < 0.05). There was also a significant increase in expression of NKp30 in CD56^bright^ cells in the HIV/AIDSWC group (*p* = 0.0275) in their absolute number. This difference was not observed in percentage (Table 4). The HIV/AIDS and HIV/AIDSWC groups also showed a significant difference in the frequency of CD56^bright^ cells expressing NKp30 in absolute number between themselves (*p* < 0.05).

### 3.5. Correlation Analysis

Correlation analysis detected a positive correlation between the expression of NKp46 and NKp30 in total NK cells (*p* < 0.0001, rs = 0.9046) and their subtypes CD56^dim^ (*p* < 0.0001, rs = 0.9297) in the HIV/AIDS group.

A positive correlation was detected between the expression of NKp46 and NKp30 in total NK cells (*p* < 0.0001, rs = 0.7580) and their subtypes CD56^dim^ (*p* = 0.0003, rs = 0.7156) and CD56^bright^ (*p* < 0.0001, rs = 0.8096) in the HIV/AIDSWC group.

A positive correlation was detected between the expression of NKp46 and NKp30 in total NK cells (*p* < 0.0001, rs = 0.7272) and their subtypes CD56^dim^ (*p* < 0.0001, rs = 0.7838) and CD56^bright^ (*p* < 0.0001, rs = 0.7121) in the healthy donor group. A positive correlation was also detected between CD4^+^ T cells and the expression of NKp46 (*p* = 0.0008, rs = 0.6241) and NKp30 (*p* = 0.0009, rs = 0.6232) in CD56^bright^ cells.

There was no correlation between the expression of NCR receptors (NKp46 and NKp30) and CD4 T cell count or CD4/CD8 ratio and infection duration in any of the patients groups studied (Table 5).

## 4. Discussion

### 4.1. Distribution of the Lymphocyte Subsets (NK and T Lymphocytes)

The hallmark of HIV-1 infection is the selective depletion of the CD4^+^ T cell caused by HIV-1 tropism by this population of cells, due to the high affinity of the protein of the viral envelope through the CD4 receptor [17] and the depletion due to permanent immune activation by inflammatory cytokines in the chronic phase of infection [18]. This is reflected in the values presented in our study, where the HIV/AIDS and HIV/AIDSWC groups differed significantly in percentage and absolute values in comparison to the healthy group (*p* < 0.0001 and *p* = 0.0002, resp.). However, according to Horberg et al. [19], treatment with HAART increased the CD4^+^ count in patients with HIV/AIDS; this was not reflected in our study. In contrast, the proportion of CD8^+^ T cells increased after HIV/AIDS infection in both groups in percentage and absolute number in comparison to the healthy group (*p* < 0.0001 and *p* = 0.0007, resp.). This increase is resonant with the findings of Naranbhai et al. [20] when studying the lymphocyte expansion in HIV-1 patients. It is important to mention that the use of certain HAART can directly affect the distribution of these cells, as demonstrated in the study of Hunt et al. [21], where the use of a drug called Maraviroc® did not affect the cell recovery rate of CD4^+^ T cells but increased CD8^+^ T cells counts in peripheral blood.

The percentage and absolute values of the distribution of NK cells found by our study in the lymphocyte segment were compatible with the study by Alter et al. [22]. These authors observed no significant differences in all NK cells, including subsets (CD56^dim^ and CD56^bright^). In this same study, the total percentage of NK cells observed in patients with acute infection proved to be significantly increased. However, in our study, the majority of individuals in the chronic stage of HIV/AIDS were studied. Together, these data suggest that the population of NK cells is usually expanded during acute infection, returning to similar levels of those observed in noninfected individuals [20, 22].

Since the advent of HAART, researchers have shown different degrees of recovery distribution in lymphocyte subpopulations, which may explain the lack of significant differences in the distribution of NK cells (and their subtypes) [23, 24] as well as lymphocyte T cells [19]. In contrast, Tarazona et al. [25] found a selective reduction of the subtype CD56^dim^ and preservation of the subtype CD56^bright^. Other authors also observed reduction in the distribution of NK cells [26–28]. This reduction appears to be partially attributable to the emergence of a novel subset of NK cells that is rare in healthy individuals, CD3^−^CD56^−^CD16^+^ NK cells that were described during chronic infections by HIV/AIDS [29]. These cells express a similar receptor profile for CD56^dim^ NK cells but are relatively cytotoxic and do not secrete cytokines. Due to the lack of appropriate markers, this subpopulation cannot be tested in our study.

### 4.2. Expression of CD336 (NKp44)

The NKp44 expression is only induced in activated NK cells and has been shown to be overexpressed in HIV+ patients with low levels of CD4^+^ T cells [13]. In our study, the expression of NKp44 in the studied cells does not show considerable levels (data not shown). More thorough* ex vivo* studies may assess if there are abnormalities in the expression of this receptor in these cells. However, Vieillard et al. [13] and Fausther-Bovendo et al. [30] observed an increase in NKp44 expression in HIV+ patients. In contrast, De Maria et al. [12] and Marras et al. [31] observed a decrease of the NKp44 functioning in HIV-1 infection and making the analysis divergent.

There is evidence that suggests that NK cells may be involved in the depletion of CD4^+^ T cells. It is known that, after infection by HIV-1, a significant fraction of CD4^+^ T cell subset expresses NKp44L, an activation ligand of the NKp44 receptor that is induced by a HIV gp41 peptide [13]. The NKp44L expression makes CD4^+^ T cells sensitive to lysis by NK cells process. This expression is strongly correlated with the decline in CD4^+^ cell count and increase of viral load [13]. The NKp44L function in the depletion of CD4^+^ T cells was confirmed in a study by Vieillard et al. [32]. Ward et al. [33] suggest that HIV-1 has acquired the ability to use NK cells to disarm the host immune system, triggering the selective killing of uninfected CD4^+^ T cells.

The activation of NKp44 can be induced by tumor cells [32]. Some studies showed that NKp44 and other NCRs usually have an important role in the lysis mediated by NK cells in various tumors, including carcinomas, melanomas, neuroblastomas, myeloid and lymphoblastic leukemia, multiple myeloma, and B cells transformed by the Epstein-Barr virus [34].

### 4.3. Decreased Expression of CD335 (NKp46) and CD337 (NKp30)

NKp46 is expressed by NK cells that are either activated or deactivated by sending activating intracellular signals by association with the CD3*ζ* chain or the *γ* chain Fc*ε*RI receptor [35]. Thus, these receptors, in combination with the NKG2D, are seen as being responsible for the spontaneous cytotoxic activity of NK cells in humans against many diseases (e.g., melanomas, carcinomas, and B lymphocytes infected by the Epstein-Barr virus). In general, the level of NKp46 expression is directly correlated with the degree of cytotoxicity of NK cells [36]. The receptor NKp30 is selectively expressed on NK cells and is associated with the dendritic cells. Human dendritic cells express the ligand for NKp30, but this ligand is unknown until now [37]. This ligand also mediates the NK-DC interaction resulting in the activation of DC or their death limiting the supply of dendritic cells [37].

Our study found a decreased frequency of NKp46 (*p* = 0.0012) and NKp30 (*p* = 0.0144) in NK cells in the HIV/AIDS group. Previous studies have also sought to assess the overall expression of NCR receptors in HIV+, showing that HIV-1 leads to an overall decrease of NCRs [11–13]. According to De Maria et al. [12], there was a significant decrease in expression of NKp46 and NKp30 receptors on NK cells in HIV+ patients. The study of Frias et al. [11] also supports this analysis, when there was a lower expression of NKp46 and NKp30 receptors in NK cells of HIV+ patients.

HIV-1 also induces functional changes in the activation of NK cells [12, 14]. For example, the activation with the K562 cell line was shown to reduce expression of CD16 and suppress the ability of NK cells to respond to additional stimulation [38]. These data suggest that NK cells in HIV-1 patients,* in vitro*, have a considerable decrease in NCR activation, which is consistent with a decrease in expression of NKp46 and NKp30 molecules observed in NK cells in peripheral blood. The reduced expression of NCR in NK cells found in our study could be responsible, at least partially, for decreased lytic activity against the virus or tumor cells. These results suggest that modulation of receptor expression on NK cells may play a role in the pathogenesis of HIV-1 and provide new insights into immunologic alterations in advanced stages of the disease [35].

Our study found a partial recovery in the expression of NKp46 and NKp30 receptor in NK cells in the HIV/AIDSWC group, which, despite being lower than in the group of healthy donors, was not statistically significant. It is known that the release of cytokines in the tumor microenvironment can induce the expression of these receptors. Different cells can participate in this modulation, such as Th17. Studies show that these cells can directly mediate the antitumor response through the effector cell recruitment to the tumor microenvironment [15, 16]. One way is through IL-17 produced by these cells which acts synergistically with the IFN inducing the production of chemokines CXCL10 and CXCL9 by malignant cells and macrophages, which induces the cell traffic that conducts antitumor functions [39]. Moreover, the ability of NK cells is increased in the presence of IL-17, since this increases cytokine expression of cytotoxic molecules such as TNF, IFN-*γ*, perforins, and granzymes B and NK cell activation receptors (NKp46, NKp44, NTB-A, and NKG2D) [40].

Another possibility for our results is the fact that the most common malignancies in the HIV/AIDSWC group induce the production of IL-12. Previous studies developed in cell culture have demonstrated that IL-12 has induced the expression of these receptors [41, 42]. It is known that the production of cytokines such as IL-15 and IL-18 have similar effects and may also act in the activation and subsequent expression of NKp46 [43, 44]. The various cancers and oncoviruses associated with HIV-1 (which is a majority of the tumor manifestations found in our group with HIV/AIDSWC) are capable of overactivating such inflammatory responses by the secretion of these proinflammatory cytokines and chemotaxis and extravasation of lymphocytes by the site of infection [45, 46].

Our data suggest that although the cancer and its associated inflammatory processes stimulate the expression of NKp30 and NKp46 in NK cells in chronic conditions of HIV/AIDS, this expression leads to a less severe manner, keeping these receptors expressed at a subnormal level but higher than the HIV/AIDS patients without cancer. These preliminary findings increase the interest in the role of NK cells and its receptors in conducting a cancer process and the history of infection, considering that the expressions of NCRs decrease along the progression of the disease [12]. It is possible that during this HIV/AIDS progression the increase in these NCRs represents an immunological indicative of cancer and other systemic inflammatory conditions.

In addition, the NCR expression in cancer patients at normal or subnormal levels can be crucial to the survival rates of the patient, because it is known that the expression of these receptors is also associated with a good prognosis [47]. Our study design did not allow us to analyze the prognosis of these patients. In this respect, we suggest other longitudinal studies to better understand the differences in the distribution of these receptors and allow monitoring of these changes over a longer period in order to relate changes in NCRs with the incidence of cancers or prognosis of cancer group to relate it to the expressions of NCRs. Many aspects need to be clarified in future studies. For example, measurements of inflammatory markers and immune activation of NK cells need to be made to actually establish a factual influence of the tumor microenvironment and to determine the implications of such systemic exposure in NK cells with the significant increase of the NCRs in the HIV/AIDSWC group when compared to the HIV/AIDS group.

Regarding the influence of HAART use in the expression of receptors, Michaëlsson et al. [47] analyzed the frequency of NKp30+ and NKp46+ cells in patients with and without treatment. Although the monitoring was only one year, they found no clear differences between the patients who had been treated and those who had not. Frias et al. [11] also did not obtain a direct relationship between the use of HAART and the recovery of these receptors. However, the same study noted that the period with an undetectable viral load was related to increased recovery of NKp30 and NKp46. This finding suggests that although long-term use of HAART does not induce a direct recovery of NCRs, more prolonged use may lead to the restoration of the innate immunity and consequently viral suppression, and in the long-term in patients older than 85 months at load, an “undetectable” could result in improved expression of NCRs, even at levels below that of healthy people. However, none of the patients in our study had an extended period of viral suppression in order to provide such recovery.

Through our correlation analysis, it was possible to observe an association between NKp46 and NKp30 receptors in all groups. These results were expected from previous studies including De Maria et al. [12], Wong et al. [35], and Lu et al. [40], since both receptors are constitutive NK cells, and expression levels of these receptors are subject to the similar variables and condition esplanades in our study. The only exception was in CD56^bright^ cells in HIV/AIDS patients without cancer that were not correlated (rs = 0.3303, *p* = 0.10680). It is possible that there are repercussions of HIV/AIDS in CD56^bright^, and these are poorly known.

Regarding the influence of other parameters in the expression of these receptors, our analysis allowed us to establish any correlation between these and the CD4 T cell numbers, CD4/CD8 ratio, and days of infection. These data are consistent with the findings of Frias et al. [11], which did not observe influence of these variables in the expression of NCRs.

Regarding the relationship observed between NKp46 (rs = 0.6241, *p* = 0.0008) and NKp30 (rs = 0.6232, *p* = 0.0009) receptors and CD4^+^ T cells in CD56^bright^ cells in healthy donors, it is possible that there are mechanisms of interaction between NK^bright^ cells and CD4^+^ T cells that may be related to expression of NCRs [48]. However, the forms of interaction between these cells are still poorly known, particularly the repercussions of these interactions in HIV/AIDS.

Finally, we hope that this study may contribute to understanding natural cytotoxicity receptors in patients with HIV/AIDS and cancer. It is crucial to consider that changes in NCRs may result in the history of the diseases as well as loss of efficiency in the monitoring of cancer cells by NK cells that together with the immunosuppression context of HIV/AIDS are critical in the beginning and throughout the evolution of these neoplastic diseases. We emphasize the need to study other types of receptors on NK cells such as lectin and KIR in patients with cancer and chronic HIV/AIDS to bring more depth to the subject.

### 4.4. Study Limitations

The technical limitations of this study include a lack of auxiliary markers to observe the CD56^−^CD16^+^ population (NK CD56^neg^) and the impossibility of activation of NK cells* ex vivo* (NKp44+) to assess if there are abnormalities in the expression of this receptor in these cells.

We cannot correlate the decreased expression of the NCRs with the function of the NK cell. A FACS-based cytotoxicity assay or cytokine production assessment would be extremely helpful to establish the functional consequences of NCR downmodulation.

The limitations of this study include a small sample size, a single-centered hospital-based study, and the impossibility of stratification to sex, age, cancer type, and treatment (HIV/AIDS as well as cancer).

## 5. Conclusion

There were no changes in the distribution of NK cells and their subtypes in either of the groups. Decreased expression of receptors NKp46 and NKp30 was observed in the HIV/AIDS group. The expression of NCR in NK cells in patients with HIV/AIDS and cancer was shown to be higher in HIV/AIDS patients without cancer.

More studies are needed that consider larger study groups and include the possibility of stratifying results to see if these findings are persistent in groups of different genders and treatments and, in the case of cancer patients, their cancers. It is necessary to determine if the changing balance of NK receptor expression may influence susceptibility to various diseases such as infections and cancer.

## Figures and Tables

**Table 1:** Baseline Characteristics from subjects in different groups.

	Number of females/males	Age in years^a^	Days since HIV/AIDS diagnosis^a^	Viral load HIV RNA/mL (range)^b^	CD4/CD8 ratio^a^	HAART treated	Days since cancer diagnosis^a^
Healthy donors	1/24	43 (20–65)	NA	NA	NA	NA	NA
HIV/AIDS	5/20	45 (19–69)	4437 (419–9267)	(<40–512.000)	0.71 (0.11–1.57)	Yes	NA
HIV/AIDSWC	4/17	45 (30–69)	2628 (88–8679)	(<40–113.000)	0.40 (0.04–1.35)	Yes	1800 (40–7488)

^a^The median (range) for each group is shown. ^b^Only the range for each group is shown. The HIV/AIDSWC group had eight patients with viral load classified as “undetectable.” The HIV/AIDS group had eighteen patients with viral load classified as “undetectable.” These patients are not included in the table. NA: not applicable.

**Table 2:** Treatment regimen in HIV/AIDS group and HIV/AIDSWC group.

Treatment regimen	Number of patients of the HIV/AIDS group	Number of patients of the HIV/AIDSWC group
NRTI + NNRTI + PI + FI	—	1
NRTI + NNRTI + PI	1	3
NRTI + NNRTI + II	2	—
NRTI + NNRTI	14	11
NRTI + PI	7	4
NRTI	1	2

NRTI: nucleoside/nucleotide reverse transcriptase inhibitors; NNRTI: non-nucleoside reverse transcriptase inhibitors; PI: protease inhibitor; FI: fusion inhibitors; II: integrase inhibitors.

**Table 3:** Frequency of T and NK (CD56^dim^ and CD56^bright^) cells from subjects in different groups.

	Median			Mean ± SD			*p* value (Kruskal-Wallis test)
	Healthy	HIV/AIDS	HIV/AIDSWC	Healthy	HIV/AIDS	HIV/AIDSWC	
Percentage (%)							
Total lymphocytes	35.50	35.50	30.00	33.61 ± 9.09	35.44 ± 10.44	32.39 ± 11.94	0.3617
T cells	55.20	59.40	57.70	54.48 ± 11.54	60.70 ± 9.14	57.52 ± 11.28	0.2204
CD4 T cells	53.00	38.20^a^	23.60^ab^	54.44 ± 11.12	37.91 ± 10.74	26.28 ± 14.99	**<0.0001**
CD8 T cells	36.10	53.00^a^	62.00^a^	35.26 ± 9.96	53.12 ± 10.32	60.66 ± 15.45	**<0.0001**
NK cells	11.30	9.70	11.60	13.21 ± 6.69	11.15 ± 7.08	12.93 ± 6.75	0.4699
CD56^dim^ NK subset	96.50	95.70	94.80	95.72 ± 2.94	94.23 ± 5.10	93.70 ± 5.14	0.2499
CD56^bright^ NK subset	3.70	4.50	5.10	4.41 ± 2.99	5.26 ± 4.14	6.37 ± 5.60	0.3283

Absolute numbers (cells/mm^3^)							
WBC count	7000	5500	6800	7148.00 ± 2233.85	5924.00 ± 1860.17	7261.90 ± 2789.52	0.0722
Total lymphocytes	2280	1960	2110	2256.40 ± 566.09	2007.60 ± 597.02	2341.42 ± 1152.39	0.4601
T cells	1159	1198	1314	1214.16 ± 416.83	1229.88 ± 454.55	1329.95 ± 640.95	0.8751
CD4 T cells	655	453^a^	280^ab^	665.36 ± 272.59	472.56 ± 228.92	355.42 ± 303.63	**0.0002**
CD8 T cells	369	603^a^	753^a^	430.08 ± 209.90	645.84 ± 252.61	812.38 ± 437.61	**0.0007**
NK cells	274	194	248	292.20 ± 173.04	211.76 ± 131.60	310.28 ± 235.15	0.1501
CD56^dim^ NK subset	257	185	239	281.60 ± 172.52	203.32 ± 131.54	294.76 ± 226.17	0.1817
CD56^bright^ NK subset	9	7	10	10.92 ± 7.29	7.72 ± 4.92	15.23 ± 12.39	0.1137

Percentage values represent the frequency (in percentage and absolute numbers) in median of T cells, CD4^+^ T cells, CD8^+^ T cells, and NK cells among all peripheral blood lymphocytes and the frequency of CD56^dim^ and CD56^bright^ NK cells among the NK cell population. Means and standard deviations (SD) are also shown. Significant *p* values are highlighted in boldface type. a = statistical difference to the healthy group (*p* < 0.05); b = statistical difference to the HIV/AIDS group (*p* < 0.05). Statistical comparisons made by Kruskal-Wallis (with Student-Newman-Keuls posttest).

**Table 4:** Frequency of T and NK (CD56^dim^ and CD56^bright^) cells expressing NCRs receptors in different groups.

	Median			Mean ± SD			*p* value (Kruskal-Wallis test)
	Healthy	HIV/AIDS	HIV/AIDSWC	Healthy	HIV/AIDS	HIV/AIDSWC	
Percentage (%) of NKp46							
NK cells	72.00	50.40^a^	69.50^b^	70.68 ± 22.38	48.03 ± 19.10	61.78 ± 21.24	**0.0012**
CD56^dim^ NK subset	72.50	43.00^a^	59.60^b^	70.13 ± 22.80	45.38 ± 19.75	59.69 ± 22.13	**0.0008**
CD56^bright^ NK subset	93.90	92.50	95.10	93.28 ± 5.19	92.54 ± 6.27	93.26 ± 6.51	0.7706
Percentage (%) of NKp30							
NK cells	38.00	20.50^a^	33.10	38.79 ± 17.33	24.90 ± 13.99	31.66 ± 16.25	**0.0144**
CD56^dim^ NK subset	41.00	21.30^a^	32.20	39.27 ± 15.96	26.64 ± 14.81	32.21 ± 17.22	**0.0286**
CD56^bright^ NK subset	11.80	10.30	14.40	11.44 ± 6.63	16.06 ± 14.49	15.60 ± 7.82	0.2068

Absolute numbers of NKp46 (cells/mm^3^)							
NK cells	158	90^a^	136^b^	199.04 ± 132.32	99.68 ± 74.37	167.23 ± 109.45	**0.0018**
CD56^dim^ NK subset	145	79^a^	127^b^	190.16 ± 131.71	91.04 ± 71.58	154.14 ± 106.78	**0.0019**
CD56^bright^ NK subset	8	7	10	10.24 ± 6.78	7.08 ± 4.34	14.09 ± 11.07	0.0754
Absolute numbers of NKp30 (cells/mm^3^)							
NK cells	86	38^a^	72^b^	111.68 ± 87.80	49.32 ± 41.16	80.09 ± 50.97	**0.0011**
CD56^dim^ NK subset	83	42^a^	69^b^	108.44 ± 83.95	49.52 ± 39.82	75.76 ± 48.23	**0.0022**
CD56^bright^ NK subset	1	1	2^b^	1.32 ± 1.31	1.04 ± 1.30	2.42 ± 2.20	**0.0275**

The values shown represent the percentage and absolute number in median within each cell population expressing a given receptor NCR. Means and standard deviations (SD) are also shown. Significant *p* values are highlighted in bold. a = statistical difference to the healthy group (*p* < 0.05); b = statistical difference to the HIV/AIDS group (*p* < 0.05). Statistical comparisons were made by Kruskal-Wallis (with Student-Newman-Keuls posttest).

**Table 5:** Correlation analysis between NCRs and CD4 T cells, CD4/CD8 ratio, and days since HIV/AIDS diagnosis.

	NKp30	CD4 T cells	CD4/CD8 ratio	Days since HIV/AIDS diagnosis
NK cells				
*Healthy donors*				
NKp46	**0.7272 **(**p** < 0.0001)	0.2585 (*p* = 0.2121)	−0.1408 (*p* = 0.5019)	NA
NKp30	—	0.1617 (*p* = 0.4400)	−0.0004 (*p* = 0.9985)	NA
*HIV/AIDS group*				
NKp46	**0.9046 **(**p** < 0.0001)	0.2400 (*p* = 0.2477)	0.1928 (*p* = 0.3559)	0.0677 (*p* = 0.7478)
NKp30	—	0.2821 (*p* = 0.1718)	0.3249 (*p* = 0.1130)	0.1428 (*p* = 0.4960)
*HIV/AIDSWC group*				
NKp46	**0.7580 **(**p** < 0.0001)	0.3351 (*p* = 0.1375)	0.0754 (*p* = 0.7454)	0.0948 (*p* = 0.6827)
NKp30	—	0.3677 (*p* = 0.1010)	0.2671 (*p* = 0.2417)	0.3910 (*p* = 0.0795)

CD56^dim^ NK subset				
*Healthy donors*				
NKp46	**0.7838 **(**p** < 0.0001)	0.2220 (*p* = 0.2861)	−0.1461 (*p* = 0.4859)	NA
NKp30	—	0.2208 (*p* = 0.2889)	0.0192 (*p* = 0.9273)	NA
*HIV/AIDS group*				
NKp46	**0.9297 **(**p** < 0.0001)	0.2213 (*p* = 0.2877)	0.1986 (*p* = 0.3412)	0.0962 (*p* = 0.6473)
NKp30	—	0.3037 (*p* = 0.1399)	0.3446 (*p* = 0.0916)	0.1946 (*p* = 0.3512)
*HIV/AIDSWC group*				
NKp46	**0.7156 **(**p** = 0.0003)	0.3558 (*p* = 0.1133)	0.0864 (*p* = 0.7095)	0.1039 (*p* = 0.6540)
NKp30	—	0.3571 (*p* = 0.1119)	0.3112 (*p* = 0.1696)	0.4727 (*p* = 0.0504)

CD56^bright^ NK subset				
*Healthy donors*				
NKp46	**0.7121 **(**p** < 0.0001)	**0.6241 **(**p** = 0.0008)	0.0698 (*p* = 0.7402)	NA
NKp30	—	**0.6232 **(**p** = 0.0009)	0.2029 (*p* = 0.3307)	NA
*HIV/AIDS group*				
NKp46	0.3303 (*p* = 0.1068)	0.3947 (*p* = 0.0508)	0.0571 (*p* = 0.7863)	0.2101 (*p* = 0.3135)
NKp30	—	0.0987 (*p* = 0.6386)	0.1911 (*p* = 0.3600)	0.3172 (*p* = 0.1222)
*HIV/AIDSWC group*				
NKp46	**0.8096 **(**p** < 0.0001)	0.3094 (*p* = 0.1722)	−0.0091 (*p* = 0.9687)	−0.1055 (*p* = 0.6489)
NKp30	—	0.2354 (*p* = 0.3043)	0.0524 (*p* = 0.8215)	0.0975 (*p* = 0.6743)

The numbers are shown as “rs (*p*)”. *p* < 0.05. Significant *p *values are highlighted in bold. The correlation coefficient rs > 0 shows positive correlation; rs < 0 stands for negative correlation. *p* < 0.05 and rs > 0.4 indicates the significant correlation between the two indexes. NA: not applicable.
